# Intranasal sensitization model of alopecia areata using pertussis toxin as adjuvant

**DOI:** 10.3389/fimmu.2024.1469424

**Published:** 2024-10-10

**Authors:** Yuying Liu, Jasmin Freeborn, Beanna Okeugo, Shabba A. Armbrister, Zeina M. Saleh, Ana Beatriz Fadhel Alvarez, Thomas K. Hoang, Evelyn S. Park, John William Lindsey, Ronald P. Rapini, Steven Glazer, Keith Rubin, Jon Marc Rhoads

**Affiliations:** ^1^ Division of Pediatric Gastroenterology, Department of Pediatrics, McGovern Medical School, The University of Texas Health Science Center at Houston, Houston, TX, United States; ^2^ Department of Neurology, McGovern Medical School, The University of Texas Health Science Center at Houston, Houston, TX, United States; ^3^ Departments of Dermatology and Pathology, McGovern Medical School, The University of Texas Health Science Center at Houston, Houston, TX, United States; ^4^ ILiAD Biotechnologies, Weston, FL, United States

**Keywords:** *Bordetella pertussis*, immunization, autoimmunity, myelin oligodendrocyte peptide, autoantigen, nasopharyngeal colonization

## Abstract

**Background:**

Nasopharyngeal *Bordetella pertussis* (BP) colonization is common, with about 5% of individuals having PCR evidence of subclinical BP infection on nasal swab, even in countries with high vaccination rates. BP secretes pertussis toxin (PTx). PTx is an adjuvant commonly used to induce autoimmunity in multiple animal models of human disease. Colocalization of PTx and myelin from myelinated nerves in the nasopharynx may lead to host sensitization to myelin with subsequent autoimmune pathology.

**Methods:**

C57BL/6J female adult mice were given varied doses and schedules of intranasal PTx, MOG_35-55_ antigen, or controls to test whether intranasal administration of PTx and myelin oligodendrocyte peptide (MOG_35-55_) could induce experimental autoimmune encephalomyelitis (EAE) in mice. While we observed systemic cell-mediated immunity against MOG_35-55_, we did not observe EAE. Unexpectedly, many mice developed alopecia. We systematically investigated this finding.

**Results:**

Patchy alopecia developed in 36.4% of mice with the optimized protocol. Pathology consistent with alopecia areata was confirmed histologically by documenting concomitant reduced anagen phase and increased telogen phase hair follicles (HFs) in biopsies from patches of hair loss in mice with alopecia. We also found reduced CD200 staining and increased CD3^+^T cells surrounding the HFs of mice with alopecia compared to the mice without alopecia, indicating HF Immune Privilege (HFIP) collapse. Systemic immune responses were also found, with increased proportions of activated T cells and B cells, as well as MHCII^+^ dendritic cells in peripheral blood and/or splenocytes. Finally, in mice initially exposed to intranasal MOG_35-55_ and PTx in combination, but not to either agent alone, splenocytes were shown to proliferate after *in vitro* stimulation by MOG_35-55._ Consistent with prior investigations, PTx exhibited a dose-response effect on immune cell induction and phenotype, with the lowest PTx dose failing to induce autoimmunity, the highest PTx dose suppressing autoimmunity, and intermediate doses optimizing autoimmunity.

**Conclusions:**

We propose that this is the first report of an autoimmune disease in an animal model triggered by colocalization of intranasal PTx and autoantigen. This model parallels a natural exposure and potential intranasal sensitization-to-pathology paradigm and supports the plausibility that nasopharyngeal subclinical BP colonization is a cause of alopecia areata.

## 1 Introduction

Animal models provide a platform to explore pathophysiology, prevention, and treatment of human autoimmune disease. Autoimmune models may be spontaneous, requiring genetic manipulation, with some requiring additional antigenic exposure. Alternatively, induced models of autoimmunity are diverse, and include adoptive transfer of immune cells or autoantibodies, viral infection, exposure to autoantigens with or without adjuvant, and exposure to adjuvant alone (1). Models that more closely replicate natural conditions may provide insight into environmental contributions to human autoimmune disease.

Two of this study’s authors previously proposed that autoimmune diseases might be caused by subclinical *Bordetella pertussis* (BP) colonization of the human nasopharynx (2–4). Subclinical BP infections are vastly more prevalent than reported cases of whooping cough (5). In multiple countries with high BP vaccination rates, evidence of mucosal subclinical BP infection is demonstrated in 4.8 – 7.1% of asymptomatic individuals by nasal swab polymerase chain reaction (PCR) or culture (6–8), and in 6.6 – 14.1% by serology (9–11). In this paradigm of autoimmunity, subclinical nasopharyngeal colonizing BP infections secrete pertussis toxin (PTx), a potent adjuvant administered by injection in many animal models of human autoimmune disease. As proposed, once PTx is colocalized with autoantigens, antigen sensitization occurs with subsequent pathology upon antigen re-exposure and host autoimmune responses (2–4).

PTx affects immunity through the innate and adaptive immune systems. PTx activates antigen presenting cells (APCs) (12, 13), enhances immunoglobulin (IgG1 and IgE) responses (14) and heightens T helper cell cytokine production (15, 16). PTx-mediated autoimmune disease models have been published in thousands of papers, including models of experimental autoimmune encephalomyelitis (EAE) — the animal model of multiple sclerosis (17, 18), experimental autoimmune uveoretinitis (EAU) (19–21), experimental autoimmune neuritis (EAN) (22), and others (23). The adjuvant effect of PTx autoimmunity is dose dependent. Lower doses may not provide sufficient adjuvant-mediated antigen sensitization, while higher and repeated doses of PTx may reduce or inhibit autoimmune disease by multiple mechanisms, including upregulation of regulatory cytokines such as IL-10 and TGF-β and by expansion of CD4^+^CD25^+^ T regulatory (Treg) cells (23–29). PTx-mediated pathology thus requires an intermediate dose and exposure frequency to optimize antigen sensitization, immune activation, and autoimmune pathology.

Alopecia areata (AA) is an autoimmune disease characterized by inflammation of hair follicles (HFs), reduced anagen (growth phase) HFs, and nonscarring hair loss. It affects nearly 2% of the population without gender bias, with symptoms ranging from patchy to complete hair loss (30). While the causes of AA are not fully understood, risk factors include specific genetic loci and the development of other illnesses, particularly allergic and autoimmune diseases. In this study, we hypothesized that colocalization of PTx (derived from BP) and myelin oligodendrocyte glycoprotein (MOG) peptide 35-55 (MOG_35-55_) at the nasopharyngeal mucosa would facilitate host sensitization and lead to an autoimmune phenotype. While initially targeting the induction of EAE-like pathology by colocalizing intranasal PTx with MOG_35-55_ to reproduce colocalization of subclinical BP and myelinated nerves of the nasopharynx (e.g., A-delta fibers) (31), we were surprised to elicit PTx-MOG_35-55_-initiated and immune-mediated alopecia. We note that previously, in a murine model of CD8^+^ clonal T-lymphocyte mediated AA, a T cell receptor was identified with dual targets: one for MOG antigen and one for the hair follicle. Nearly all transgenic mice expressing this T cell receptor developed AA (32).

To our knowledge, the experiments and evidence presented herein are the first report of autoimmune disease triggered by colocalization of intranasal autoantigen and PTx adjuvant. This model supports the plausibility that nasopharyngeal subclinical BP colonization is a potential cause of AA.

## 2 Materials and methods

### 2.1 Animals

Female wild-type (WT) C57BL/6J (10-week-old) mice (# 000664) were purchased from Jackson Laboratories (Bar Harbor, ME) and allowed to acclimatize for 2 weeks before experimentation. Mice were housed in groups in polycarbonate cages with free access to a standard diet and water in the specific pathogen free (SPF) animal facility at the University of Texas Health Science Center at Houston. This study was carried out in accordance with the recommendations of the Guide for the Care and Use of Laboratory Animals (NIH) and the Institutional Animal Care and Use Committee (IACUC) of The University of Texas Health Science Center at Houston. The protocol was approved by the IACUC (protocol numbers: AWC-18-0051 and AWC-21-0110).

### 2.2 Intranasal administration of MOG_35-55_ and PTx

#### 2.2.1 Mouse handling for intranasal administration

The handling procedure for intranasal administration of MOG_35-55_ and PTx in PBS solution to non-anesthetized mice was based on a previously described protocol (33) with modification. Two research investigators coordinated intranasal administration, with one holding the mouse and the other delivering the agent, instead of using a more immobilizing grip by a single operator who also administered the reagents. By doing so, our procedure reduced the weeks of acclimation needed for mouse immobilization and reduced the amount of stress to which the mice were exposed.

#### 2.2.2 Reagent sources, preparation, and intranasal delivery volume

MOG_35-55_ purchased from AnaSpec Inc. (Fremont, CA) was dissolved in PBS with the stock concentration of 10 mg/mL. PTx was purchased from Sigma (St. Louis, MO) with the stock concentration of 0.2 mg/mL. The working concentrations of MOG_35-55_ or PTx were adjusted based on the experimental design in the expected volumes. The volume for intranasal delivery was 10 μL per nostril by using a 20 μL pipettor and gel loading pipette tip. The use of PTx in the experiments was approved by Chemical Safety Committee (protocol numbers: CSC-15-021, and CSC-21-045) of the University of Texas Health Science Center at Houston.

### 2.3 Experimental design and treatment protocol

**Figure 1:**
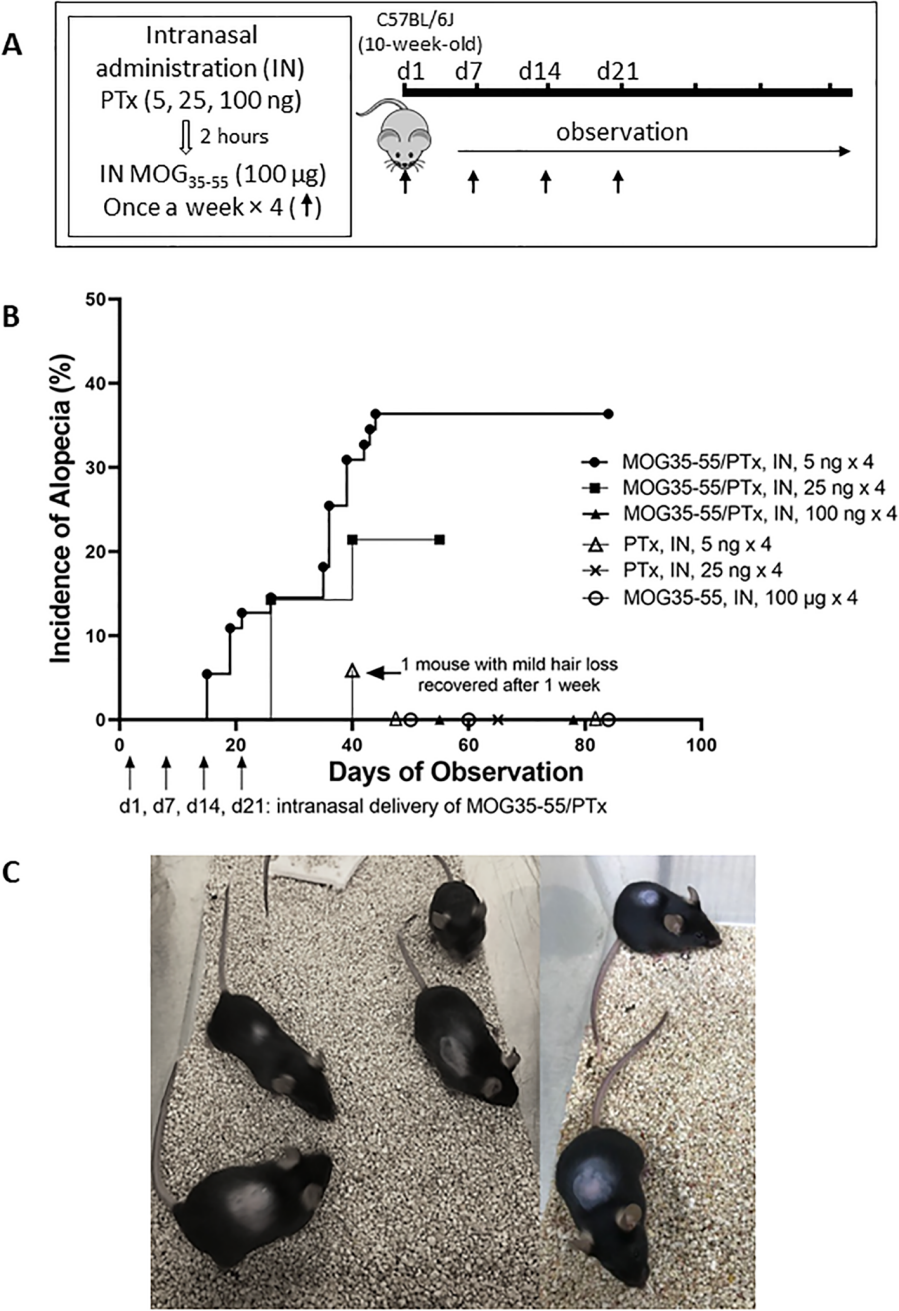
Development of autoimmune alopecia in mice after repeated intranasal administration of PTx and MOG_35-55_. **(A)** Experimental protocol for inducing alopecia. **(B)** The onset of alopecia and % developing alopecia during the observation period. Mouse numbers in each group are as follows: MOG_35-55_/PTx, intranasal, 5 ng × 4, n = 55; MOG_35-55_/PTx, intranasal, 25 ng × 4, n = 14; MOG_35-55_/PTx, intranasal, 100 ng × 4, n = 10; PTx, intranasal, 5 ng × 4, n=17; PTx, intranasal, 25 ng × 4, n = 5; MOG_35-55_, intranasal, 100 μg × 4, n = 17. Each dot represents several mice with or without alopecia on the observed indicated dates. **(C)** Representative images are shown for mice with alopecia areata.

We administered different dosages of PTx and varied the number of intranasal administrations to test the ability of PTx to promote autoimmunity, initially intended to induce EAE, as indicated in **Supplementary Figure 1**. In Experiment I, each mouse was intranasally administered an initial (1^st^) dose of a mixture of PTx (100 ng or 200 ng or 400 ng) and MOG_35-55_ (250 μg) on d1. At d2 (post-16h of 1^st^ dosage), each mouse was intranasally administrated a 2^nd^ dose of PTx alone (100 ng or 200 ng or 400 ng, respectively, without MOG_35-55_). For the PTx dosage of 200 ng × 1, each mouse was intranasally administered only one dosage of a mixture of PTx (200 ng) and MOG_35-55_ (250 µg). In Experiment II, each mouse was administered a mixture of intranasal PTx (200 ng) and MOG_35-55_ (250 μg) at d1, d7, d14, and d21 (×4) or at d1, d4, d7, d10, d14, d17, d21, d24 (×8). In Experiment III (**Figure 1A**), each mouse was initially administered intranasal PTx (5 ng, 25 ng, 100 ng, respectively), and 2 hours later mice were administered intranasal MOG_35-55_ (100 μg) at d1, d7, d14, and d21 (×4).

For each experiment, after the last intranasal administration, the mice were observed daily for at least 3 weeks. No EAE phenotype was observed in any experimental designs by the end of the experiment. However, we recorded the incidence of alopecia, and collected skin biopsies for histological evaluation the same day that we observed the onset of alopecia. At the end of the experiment, we collected blood and spleen samples, isolated splenocytes for *in vitro* MOG_35-55_ stimulation, and analyzed immune cell markers.

### 2.4 Splenocyte isolation and *in vitro* cell proliferation assay

Single cell suspensions from the spleen were prepared by gently fragmenting and filtering the tissues through 40 μm cell strainers (BD Bioscience, San Jose, CA) into RPMI 1640 complete medium (Sigma-Aldrich, St. Louis, MO) followed by removal of red blood cells with ACK lysis buffer (Quality Biologicals, Gaithersburg, MD). For *in vitro* stimulation, splenic lymphocytes isolated from each mouse were performed in triplicate with a total n = 5 – 15 mice per group. Briefly, 5 × 10^5^ cells were plated into 24-well cell culture plates, and cells from each mouse were stimulated with 10 μM of MOG_35-55_ (+MOG) or PBS (-MOG) for 3 days, followed by colorimetric measurement for cell proliferation using the tetrazolium dye, 2,3-bis (2-methoxy-4-nitro-5-sulfophenyl)-5-[(phenylamino) carbonyl]-2H-tetrazolium hydroxide (XTT) according to the manufacturer’s protocol (TACS™ XTT Cell Proliferation/Viability Assay, R & D System, Minneapolis, MN) (34), as described previously (35). We calculated the percentage of cells proliferating as follows: [(OD_+MOG_-OD_-MOG_)/OD_-MOG_] × 100.

### 2.5 Histopathology and immunohistochemistry staining to evaluate autoimmune alopecia in skin biopsies

#### 2.5.1 Skin biopsies

Skin biopsies were performed using a sterile Integra Miltex 1.5 mm disposable punch biopsy (Integra Life Science Production Corporation, Mansfield, MA) while the mouse was anesthetized with isoflurane in the presence of oxygen in an induction chamber for 3 – 4 minutes before moving the mouse to a nose cone attached to isoflurane and an oxygen source. In addition, bupivacaine (0.25%) was injected subcutaneously (< 1 mg/kg, 20 – 30 μL) around the biopsy site before the skin biopsy.

#### 2.5.2 H&E and IHC for CD3 and CD200

The skin tissues were fixed in formalin, processed, and stained with hematoxylin and eosin (H&E). IHC staining of CD3^+^ T cells was performed with a Dako Omnis system (Agilent, Santa Clara, CA) and Agilent CD3 polyclonal antibody with high pH antigen retrieval. CD3^+^ T cell staining was performed by the Histology Laboratory of the Department of Pathology and Laboratory Medicine of the University of Texas Health Science Center at Houston. C57BL/6J mouse thymus tissue was used as positive staining control for CD3 staining. For IHC of CD200, proteinase K (Abcam AB64220, Waltham, MA) was used for enzymatic antigen retrieval, followed by blocking endogenous peroxidase by 3% H_2_O_2_ (Sigma-Aldrich) and non-specific binding by 2.5% goat serum (Vector Laboratories, Newark, CA). Rabbit monoclonal anti-mouse antibody CD200/OX2 (AbCam AB314662) was used for staining at 1:500 dilution overnight at 4°C; secondary antibody treatment was with ImmPRESS HRP Goat Anti-Rabbit IgG Polymer Detection Kit (Vector Laboratories MP-745) for 30 min at room temperature (RT), followed by addition of DAB Substrate (Vector Laboratories, SK-4105) and counterstaining with hematoxylin.

#### 2.5.3 Analysis and quantification

For H&E staining sections, we analyzed the numbers of telogen and anagen phase follicles and quantified counts at 100× magnification at 3-5 image fields for each tissue section with ImageJ (FIJI) (36) software (NIH). We calculated the % of anagen or telogen phase follicles among all HFs in each field and calculated the mean ± SD (%) for mice with alopecia compared to mice with no alopecia. For IHC CD3 and CD200, we compared positively stained cells in skin biopsies of mice with alopecia compared with mice without alopecia. We also analyzed the numbers of HFs with CD200^+^stained in the epithelium and quantified counts at 200× magnification in at least 5 image fields to calculate the % of HFs with CD200^+^stained epithelium among all HFs in each field and calculated the mean ± SD (%) for mice with alopecia compared to mice with no alopecia.

### 2.6 Staining immune cells for flow cytometry

To evaluate the activated T cells and B cells in the mice, as well as MHCII^+^ APCs in the splenocytes and circulating peripheral blood mononuclear cells (PBMCs), cells were surface-stained by fluorochrome-conjugated mouse antibodies. Specifically, we used antibodies including CD3 (17A2) conjugated with brilliant violet (BV)421, CD19 (6D5) conjugated with fluorescein isothiocyanate (FITC), GL-7 (GL7) antigen (also called Ly77, a T cell and B cell activation marker) conjugated with phycoerythrin/Cyanine7 (PE/Cy7), CD11c (N418) conjugated with Alexa Fluor 700 (AF700), CD11b (M1/70) conjugated with peridinin-chlorophyll proteins/Cyanine5.5 (PerCP/Cy5.5), and MHCII (10-3.6) conjugated with PE. All conjugated antibodies were purchased from BioLegend (San Diego, CA). MACS buffer consisting of phosphate-buffered saline, 0.5% bovine serum albumin (Hyclone GE Life Science, Logan, UT), and 2 mM EDTA (Lonza, Bethesda, MD) was used for washing the cells. Prepared samples were analyzed by flow cytometry using a BD LSRFortessa Flow Cytometer (BD Bioscience, San Jose CA), and data were analyzed with FlowJo software (FlowJo, BD, Ashland, OR).

### 2.7 Statistical analysis

Significance was determined using one-way ANOVA for multiple comparisons with Tukey post-hoc tests. An unpaired t-test was used to determine significance between the means of two groups. The statistical analysis was performed using GraphPad Prism version 9.4.1 (GraphPad Software, San Diego, CA). Data are represented as means ± SD. Values with p < 0.05 were considered statistically significant.

## 3 Results

### 3.1 Alopecia was induced by intranasal administration of MOG_35-55_ and PTx

Mice with intranasal administration of lower dosage ranges of PTx including 5ng, 25ng, and 100ng, respectively, in combination with intranasal administration of MOG_35-55_ at 100 µg, dosed once a week for 4 weeks were observed for alopecia (**Figure 1A**, **Supplementary Figure 1** Experiment III). The incidence of alopecia was 36.4% (20/55) for the mice that received MOG_35-55_/PTx 5 ng × 4 and 24.2% (3/14) for the mice that received MOG_35-55_/PTx 25 ng × 4; while mice that received MOG_35-55_/PTx at 100 ng × 4, as well as PTx only or MOG_35-55_ only did not develop alopecia (**Figure 1B**). It was noted that in the PTx only group, one mouse showed hair loss on d40 post-initial (1^st^) intranasal administration, but, unlike all other mice that developed alopecia, hair growth resumed just one week later (**Figure 1B**).

During the trials of different dosages and treatment protocols for intranasal MOG_35-55_/PTx (**Supplementary Figure 1**), we observed that lower dosages of PTx (5 ng × 4, and 25 ng × 4) induced hair loss as early as d14 following the initial (1^st^) intranasal administration (**Figures 1B, C**). At the end of the observation period, 25% (5/20) of mice with alopecia demonstrated mild-moderate hair regrowth (**Supplementary Figure 2**).

### 3.2 Increased telogen and reduced anagen phase hair follicles in skin biopsies were observed in mice with alopecia

**Figure 2:**
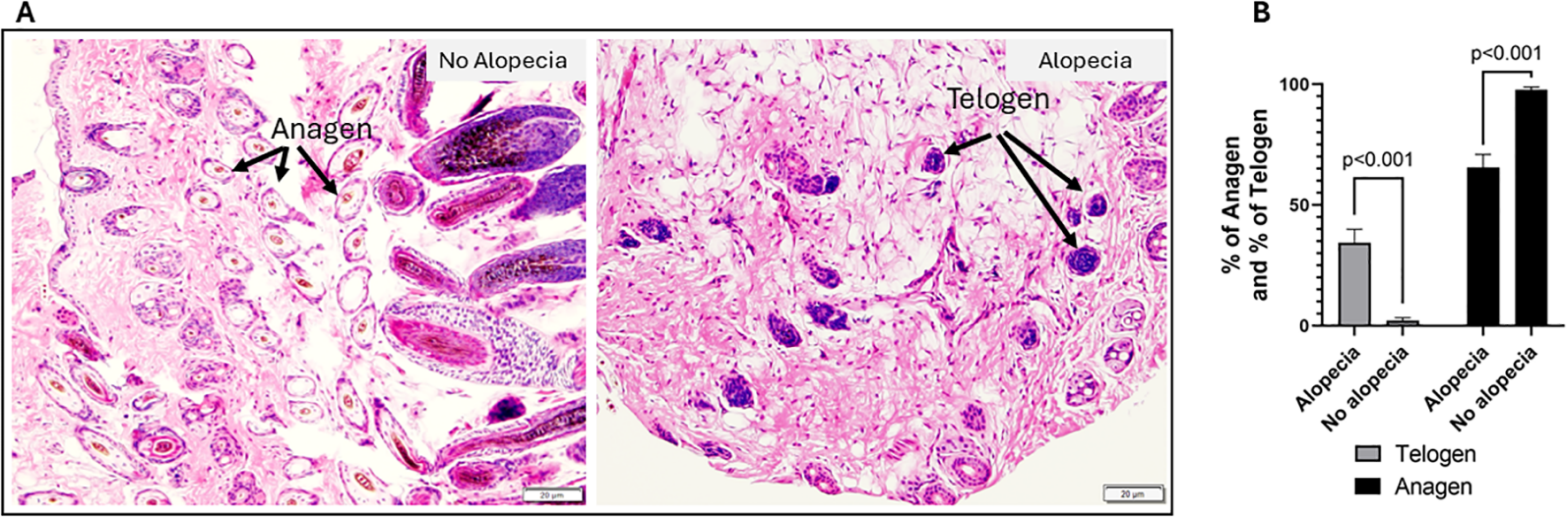
Histological evaluation of autoimmune alopecia with mouse skin biopsies. **(A)** Representative H&E-stained images. Images in the left panel are from mice with no alopecia, the arrows indicate anagens. Images in the right panel are from mice with alopecia; the arrows indicate telogens. 100 × magnification. **(B)** % of anagen and % of telogen follicles in mice with alopecia compared to those without alopecia. Significant p values indicated in the figure.

One of the features of AA in human and other mouse models has been a shift from anagen to telogen phase and anagen-like nanogen type HFs with no central hair shaft (37, 38). We evaluated the anagen and telogen HFs in the histological biopsies stained by H&E (**Figure 2A**). The results showed significantly increased telogen and reduced anagen phase follicles (**Figures 2A, B**) in mice with alopecia compared to mice with no alopecia. The percentage of telogen phase follicles in the microscopic field at 100× magnification increased from 2.2 ± 1.0% in no alopecia mice to 34.5 ± 5.4% in mice with alopecia; while the percentage of anagen phase follicles was reduced from 97.8 ± 1.1% in those without alopecia to 65.5 ± 5.5% in mice with alopecia (all p < 0.001, **Figure 2B**).

### 3.3 Mice with induced alopecia demonstrated altered hair follicle immune privilege markers

**Figure 3:**
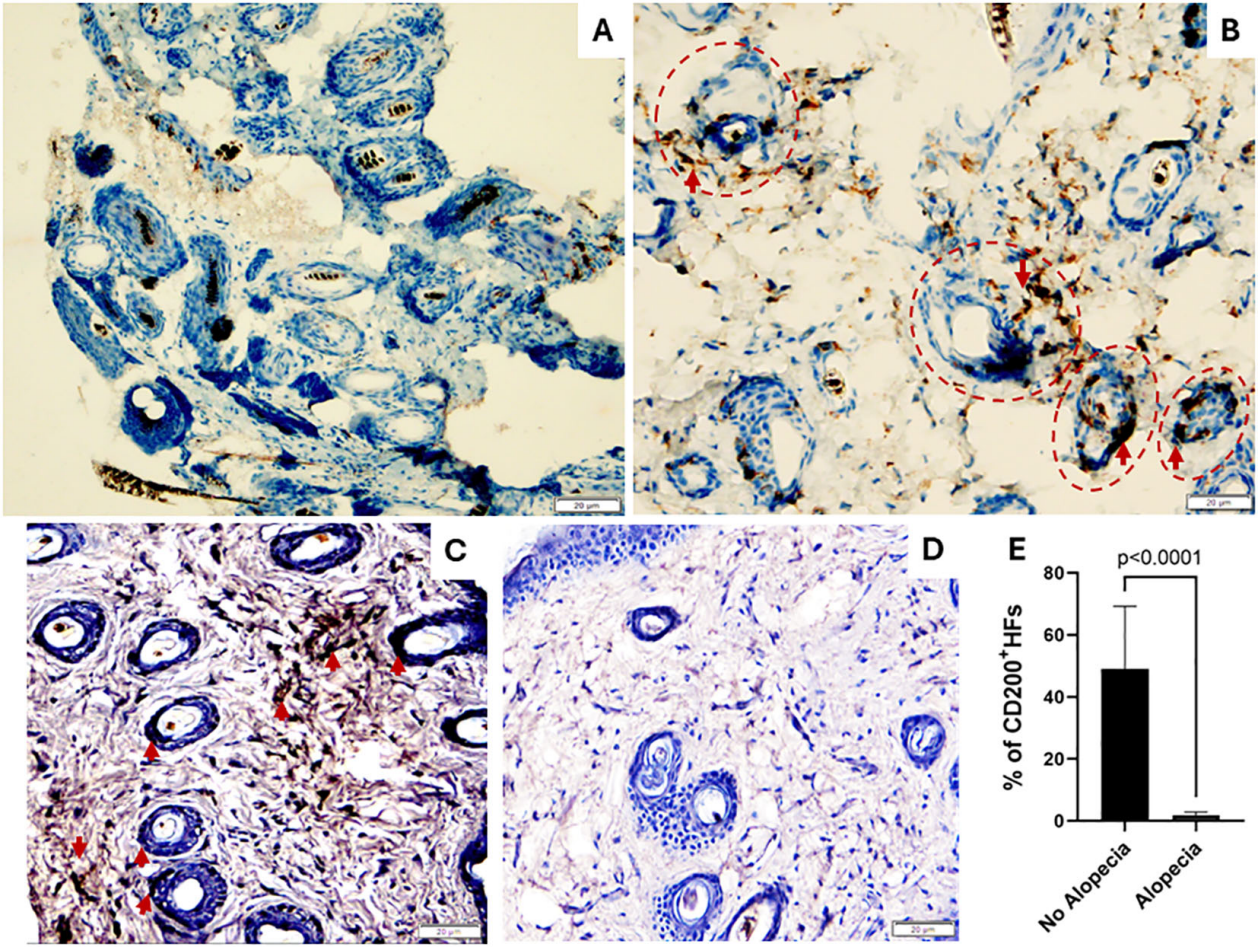
Skin biopsies: CD3 and CD200 expression. **(A, B)** Images show increased CD3^+^ T lymphocytes around the hair follicles (HFs, Red Circles) in mice with alopecia **(B)** compared to normal skin biopsies **(A)**. **(C-E)** Images show CD200 is highly expressed hair follicle epithelium and dermis around HFs in normal mice without alopecia **(C)** but diminished in those with alopecia **(D)**. **(E)** % of # of HFs with CD200^+^stained epithelium in mice with alopecia compared to those without alopecia. 200 × magnification. Significant p values indicated in the figure.

HFIP is a dynamic process maintained by several mechanisms resulting in immune tolerance and suppression of immune-mediated inflammation. Among several mechanisms believed to support HFIP, a CD200-CD200R interaction is thought to promote tolerance and prevent autoimmunity within the epidermis and dermis around the HFs, which is often associated with fewer T cells. The connective tissue sheath is thought to guard against immune cell infiltration by generating a proteoglycan barrier during the anagen phase (39). Previous studies showed CD200 expressed on the epithelial cells on murine HFs as an indicator of tissue-specific tolerance (40). Therefore, we performed immunohistochemistry staining of skin biopsies with anti-CD3 and anti-CD200 antibodies. We observed increased CD3^+^ T cell infiltration around HFs in skin biopsies of mice with alopecia (**Figure 3B**) but not in mice without alopecia (**Figure 3A**). In addition, we found CD200^+^staining on epithelium of HFs and around HFs in the mice with no alopecia (**Figure 3C**) and diminished CD200^+^staining of epithelium of HFs and around HFs in the mice with alopecia (**Figure 3D**). The percentage of HFs with CD200^+^stained epithelium in the mice with no alopecia (49.1 ± 20.1%) was significantly higher than that in the mice with alopecia (1.8 ± 1.0%) (p < 0.0001, **Figure 3E**). The results support HFIP collapse in mice with induced alopecia, consistent with findings in alopecia areata.

### 3.4 Mice with induced alopecia demonstrated systemic immune activation

#### 3.4.1 Circulating activated T lymphocytes

**Figure 4:**
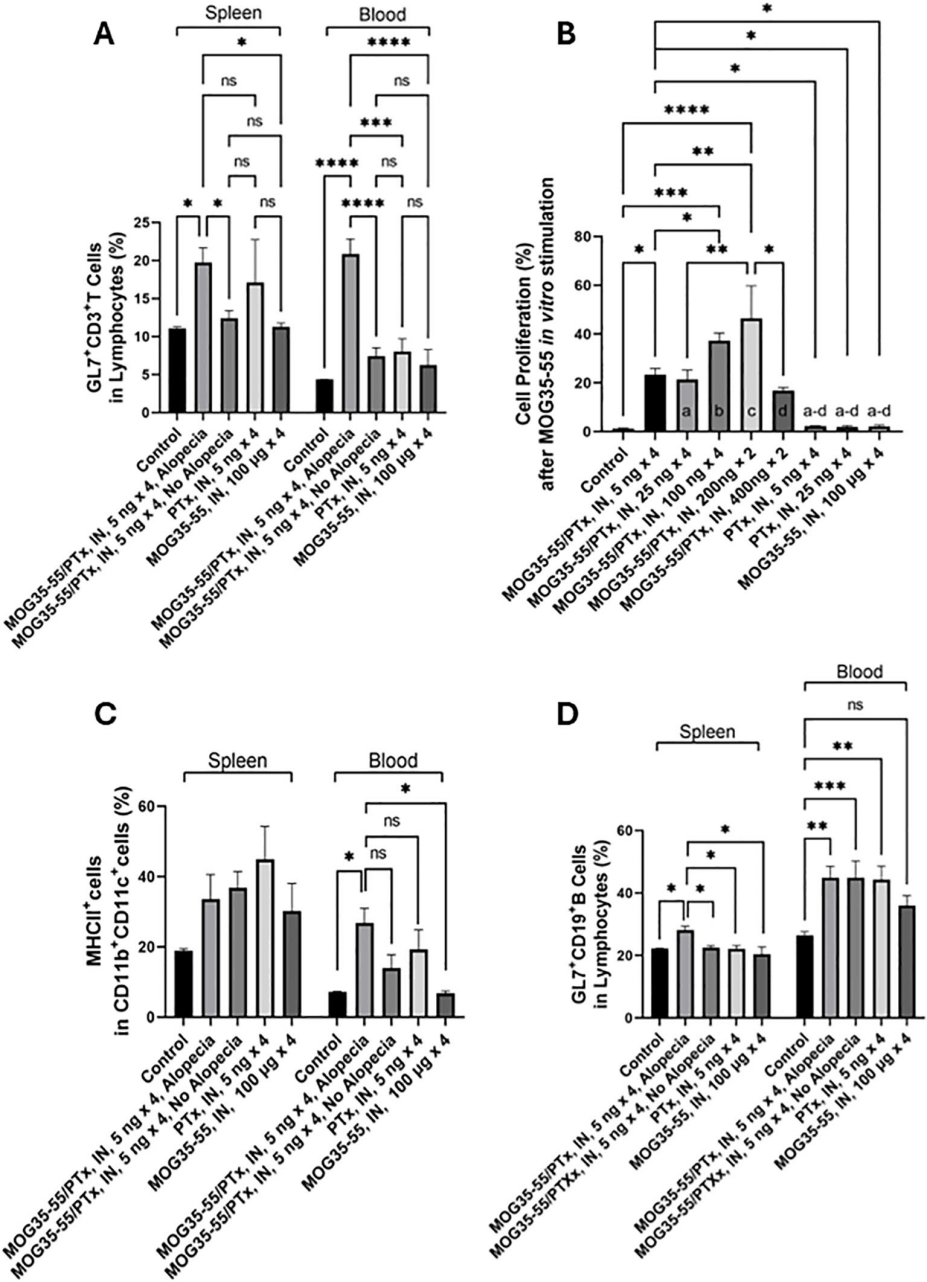
Systemic immune activation. **(A)** The percentage of GL7^+^CD3^+^ T cells (indicating activated T cells) among lymphocytes in the spleen and blood of mice with different intranasal exposures. **(B)** XTT spectrophotometric absorbance, representing cell proliferation after splenocytes were stimulated *in vitro* with 10 μM MOG_35-55_ for 72h. Splenic lymphocytes isolated from each mouse were studied in triplicate from n = 5-15 mice per group. Only groups with significant differences are shown; a-d indicates the significance of difference with group comparisons: a, groups *vs.* MOG_35-55_/PTx, intranasal, 25 ng × 4; b, groups *vs.* MOG_35-55_/PTx, intranasal, 100 ng × 4; c, groups *vs.* MOG_35-55_/PTx, intranasal, 200 ng × 2; and d, groups *vs.* MOG_35-55_/PTx, intranasal, 400 ng × 2. **(C)** The percentage of MHCII-expressing cells among CD11b^+^CD11c^+^ antigen-presenting cells in the spleen and blood of mice with different intranasal treatment protocols. **(D)** The percentage of GL7^+^CD19^+^ B cells (indicating activated B cells) from the lymphocyte population in the spleen and blood of mice with different intranasal treatment protocols. N = 5-15 mice per group, *p < 0.05, **p < 0.01, ***p < 0.001, ****p<0.0001, and ns, no significant difference.

To assess immune cell activation, we focused on GL7 expression on T (CD3^+^) cells. GL7 is a marker for polyclonally activated T and B cells in mice (41, 42). We counted the activated GL7^+^CD3^+^ T cells in the spleen and circulating blood by flow cytometric analysis. CD3^+^ T cell populations were first gated from the defined lymphocyte population, and GL7^+^ activated T cells were further defined within the CD3^+^ T cell population (**Supplementary Figure 3**). We found an increased proportion of activated CD3^+^ T cells in the spleen and circulating blood of mice with alopecia compared to groups of mice without alopecia (**Figure 4A**).

#### 3.4.2 Immune cell responsiveness to MOG_35-55_ *in vitro* stimulation

To assess systemic sensitization to MOG_35-55_, we exposed splenocytes from mice in nine experimental groups to MOG_35-55_ and measured proliferation by optical density. Prior exposure conditions of these groups were those to PBS alone, MOG_35-55_ and PTx at doses of 5, 25, 100, 200 and 400 ng × 4, PTx at 5 and 25 ng × 4, and MOG_35-55_ alone (**Supplementary Figure 1**). We found that cell proliferation was significantly increased in the groups initially sensitized with the intranasal administration of combined MOG_35-55_ and PTx, compared to the control mice with no intranasal administration of PTx or MOG_35-55_, but not in the groups treated with intranasal administration of either MOG_35-55_ or PTx alone. We noted that *in vitro* cell proliferation in response to MOG_35-55_ was maximal in the groups initially sensitized with intranasal administration of the combination of MOG_35-55_/PTx with PTx at total 400 ng (200ng × 2), with reduced responses to PTx at a total of 800 ng (400ng × 2) (**Figure 4B**).

#### 3.4.3 Circulating APCs and activated B cells

MHC class II molecules are primarily expressed by APCs, such as monocytes, macrophages, dendritic cells, and B lymphocytes (43). These cells are involved in external antigen processing via MHC II with antigenic peptide presentation to CD4^+^ T helper cells. Most APCs also express CD11b and CD11c (44). Among non-T non-B cell populations, we identified CD11c^+^ and CD11b^+^CD11c^+^ cells and characterized the percentage of cells expressing MHC II (**Supplementary Figure 2**). We found a significant increase of MHC II-expressing CD11b^+^CD11c^+^ cells in the circulating blood, but not in the spleen of mice with alopecia (**Figure 4C**). Activated CD19^+^ B cells were also defined by GL7^+^ cells among CD19^+^ B cells (**Supplementary Figure 3**), and we found that the percentage of activated CD19^+^ B cells was significantly increased in the spleen of mice that developed alopecia. However, we also saw an increased proportion of activated B cells in the blood of mice that had received intranasal MOG_35-55_/PTx, regardless of the presence of alopecia. Intranasal PTx alone, but not MOG_35-55_ alone, promoted B cell activation in circulating blood (**Figure 4D**).

## 4 Discussion

In this study, we hypothesized that nasopharyngeal colocalization of the potent adjuvant PTx and a myelin component MOG_35-55_ would facilitate host sensitization to MOG_35-55_ with subsequent autoimmune pathology. We tested intranasal delivery of different dosages and treatment protocols of PTx with MOG_35-55_ and found that at a low dosage of PTx (5 ng), the combination induced large patches of alopecia. Onset occurred as early as 2 weeks following the first intranasal administration. Alopecia was maintained without hair regrowth in 75% of mice with alopecia, while 25% of alopecia mice showed hair regrowth at 12 weeks (84 days), the maximum period of observation after intranasal administration. Skin biopsies demonstrated cardinal features for AA with a phase shift from anagen to telogen follicles, increased T cells, and reduced CD200 positive staining of epithelial cells on and around the HFs. We propose that these experiments represent the first animal model of an autoimmune disease induced by intranasal sensitization to an autoantigen, with PTx acting as the sole adjuvant. We further propose that colocalization of PTx and myelin may occur naturally in humans, given the high rate of subclinical colonizing BP infections, their ability to secrete PTx into local mucosa, and the subsequent colocalization of PTx and myelin from myelinated nasopharyngeal nerves (such as A-delta fibers) (31, 45).

AA in humans is characterized by non-scarring hair loss on the scalp or any hair-bearing surface (46, 47). Its etiology is complex and multifactorial, with contributions from genetic and environmental factors. A genetic contribution is evident in that a family history of AA is noted in 0-8% of adults and 10-51% of children (30). A 55% concordance rate is observed in identical twins (48, 49). The balance of the risk is likely due to environmental factors. We propose that one such factor may be nasopharyngeal subclinical BP colonization with secretion of the potent adjuvant PTx at the local mucosa.

Alopecia can be categorized as scarring or non-scarring. Lymphocytic or neutrophilic scarring alopecia includes chronic cutaneous lupus, lichen planopilaris, and folliculitis decalvans, conditions characterized by destroyed HFs replaced by fibrous tissue. PTX/MOG_35-55_-induced alopecia in our study did not reveal fibrous tissue replacing HFs and would be categorized as non-scarring. Other non-scaring alopecias demonstrate pathology distinct from what was demonstrated in the present study. Androgenic alopecia is associated with HF miniaturization and variation in HF size, which were not seen in our study. Telogen effluvium generally shows a normal number of HFs, increased telogen phase follicles, HF miniaturization and perifollicular collagen. Trichotillomania is manifested by distortion of HF anatomy, perifollicular and intrafollicular hemorrhage, but no lymphocyte infiltration (38).

The evolution of AA is tied to the collapse of HF immune privilege (HFIP). Many potential components supporting HFIP have been proposed, including physical barriers, CD200 protective signaling, local generation of immunosuppressant cytokines (TGF-β, IL-10), downregulated MHC I-related molecules (β2-microglobulin), and low numbers of normal T cells (both CD4^+^ and CD8^+^) and natural killer cells (39, 50). We observed reduced CD200 positivity and increased T cells around HFs in mice with alopecia, indicating the collapse of HFIP, and a HF phase shift from anagen to telogen (51–54).

In human AA, scalp histology in early disease is characterized by a peribulbar immune cell infiltrate, predominately T lymphocytes and other immune cells (APCs and mast cells) (38). However, the histological features of murine AA are different. Instead of a largely peribulbar lymphocytic infiltration (38), other groups have shown that mice have a more generalized lymphocytic infiltration that extends to the distal follicle between the hair bulb and sebaceous gland (55), as observed in our study. In addition, using intranasal PTx as adjuvant, we observed diffuse CD3^+^ T cell infiltration in skin tissues of mice with alopecia. This finding is consistent with human AA, in which the presence of CD3^+^ T-cells in the dermis, subcutis, and empty follicular fibrous tracts are diagnostically supportive of AA (56, 57).

Genomic regions associated with AA include those encoding IFN-γ-mediated cytotoxicity, and others encoding T cell activation and proliferation (58–60). We observed that in addition to increased T lymphocyte dermal infiltration in alopecia-affected mice, there were increased numbers of circulating activated T and B cells associated with intranasal PTx. A recent study reported that IFN-α-producing plasmacytoid dendritic cells (pDCs) may contribute to autoimmune alopecia in mice (61). In the current study, we likewise identified a significant increase in the percentage of circulating MHCII^+^CD11b^+^CD11c^+^ dendritic cells in mice with alopecia that were sensitized with PTx and MOG_35-55_.

To investigate how MOG_35-55_ peptide may be a casual factor in autoimmune alopecia, we performed a homology search by entering its amino acid sequence into the SMARTBLAST tool provided by National Center for Biotechnology Information (NCBI). We noted that the MOG_35-55_ amino acid sequence has 52.4–57.1% homology to *selection and upkeep of intraepithelial T-cells protein isoforms and precursors* (SKINT genes), and 57.1–66.7% homology to *butyrophilin-like protein isoforms* (BTN genes). BTN genes have similar functions to SKINT genes, promoting γδ T cell formation in the fetal thymus, T cell migration to skin, T cell receptor activation, and activating inflammatory signaling (62, 63). It has been shown that BTN3A targets promote T cell cytotoxicity (64), and BTN2A is a normal ligand for the DC-Sign receptor on immature monocyte-derived DCs that promote DC maturation (65). There may therefore be cross reactivity between MOG_35-55_ and these follicular proteins, with AA pathology possibly resulting from intranasal sensitization to MOG_35-55_ and subsequent epithelial and follicular pathology due to molecular mimicry leading to a hyperimmune response at the hair follicle. A MOG-associated model of alopecia has previously been described (32). Rag1^-/-^ mice were transplanted with a 1MOG244.1 T cell receptor into CD8^+^T progenitor cells that had dual specificity for myelin and hair follicles, in this model, all mice developed alopecia (32).

To date, there have been no reports of intranasal administration of PTx and/or MOG_35-55_ as promoters of alopecia. Like many biological systems that manifest an optimal response within a range of exposures, we demonstrated a “Goldilocks” dose-dependent effect of PTx, as has been seen previously in autoimmune modeling. Specifically, PTx exhibited a dose-response effect on immune cell induction and phenotype, with the lowest (absent) PTx doses failing to induce autoimmunity, the highest PTx doses suppressing autoimmunity, and intermediate doses optimizing autoimmunity. Interestingly, in EAE mouse models, higher PTx dosing increases Treg cell numbers and upregulates neuronal vascular endothelial growth factor (VEGF) which may protect neurons, reduce lymphocyte infiltration, and decrease microglia activation (24–26, 66–68). Furthermore, in other labs higher doses of PTx (1000 ng) significantly attenuated EAE (26), and chronic repetitive dosing (300 ng weekly for 6 months) prevented EAE (25). Others established that low dose PTx (25 ng) results in reduced EAE clinical scores (66), while moderate dosing (200 – 400 ng of intraperitoneal PTx) with MOG_35-55_ induces severe EAE. This well-documented Goldilocks dosing effect in EAE is consistent with findings in the current study. While EAE was not demonstrated in this intranasal PTx-mediated sensitization model, we have not ruled out that further alterations in PTx and MOG_35-55_ dosing and timing could lead to EAE, given that systemic sensitization to MOG_35-55_ was observed.

In the current intranasal sensitization model, 36% of mice developed alopecia. Several factors may account for this incidence. There may be variability of intranasally administered PTX or MOG_35-55_ in an individual mouse, as reagents may have been expelled by mice after administration or met with varying intranasal conditions such as the amount of mucus. We may have performed our experiment on mice with suboptimal characteristics or under suboptimal conditions to evidence an autoimmune phenotype. In humans, the concordance rate of AA in monozygotic twins raised in the same household was reported at 40-50%, indicating that both genetic and environmental factors are involved in the etiology of AA (49). Differential susceptibility to disease among the same strain of mice may be partially related to epigenetic factors. For example, two genetically identical mice grown in identical conditions may have different epigenomes, such as their degree of CpG methylation, altering susceptibility to disease (69, 70).

In conclusion, our study is the first to report that colocalized intranasal PTx and MOG_35-55_ can induce an AA-like disease in mice. There are at least 20 preclinical models for psoriasis, 19 for atopic dermatitis, and 11 for vitiligo, but there are few models for AA (55, 71). Our observations support the hypothesis that colocalization of PTx and MOG_35-55_ at the nasopharyngeal mucosa may facilitate host sensitization, leading to autoimmune AA. Given the frequency of human nasopharyngeal BP colonization, the ability of PTx to act as an adjuvant, and the presence of myelinated nerves in the nasopharynx, we suggest that our intranasal model parallels a paradigm that occurs naturally in humans and could lead to human disease. This model may also provide a useful platform to further unravel the complexities of AA.

## Data Availability

The original contributions presented in the study are included in the article/**Supplementary Material**. Further inquiries can be directed to the corresponding author.
