# Supplementary material for: Intranasal sensitization model of alopecia areata using pertussis toxin as adjuvant
Source: Front Immunol. 2024 Oct 10;15:1469424. doi: 10.3389/fimmu.2024.1469424 (PMC11499204; doi:10.3389/fimmu.2024.1469424)
Supplement: Supplementary file 1 [file DataSheet1.docx]

**Intranasal sensitization model of alopecia areata using pertussis toxin as adjuvant**

**Yuying Liu^1*^, Jasmin Freeborn^1^, Beanna Okeugo^1^, Shabba A. Armbrister^1^, Zeina M. Saleh^1^, Ana Beatriz Fadhel Alvarez^1^, Thomas K. Hoang^1^, Evelyn S. Park^1^, J. William Lindsey^2^, Ronald P. Rapini^3^, Steven Glazer^4^, Keith Rubin^4^, and J. Marc Rhoads^1^**

^1^Division of Pediatric Gastroenterology, Department of Pediatrics, McGovern Medical School, The University of Texas Health Science Center at Houston, Houston, TX, USA

^2^Department of Neurology, McGovern Medical School, The University of Texas Health Science Center at Houston, Houston, TX, USA

^3^Departments of Dermatology and Pathology, McGovern Medical School, the University of Texas Health Science Center at Houston, Houston, TX, 77030, USA

^4^ILiAD Biotechnologies, Weston, FL, 33331, USA

Supplementary Material

# Supplementary Figure 1


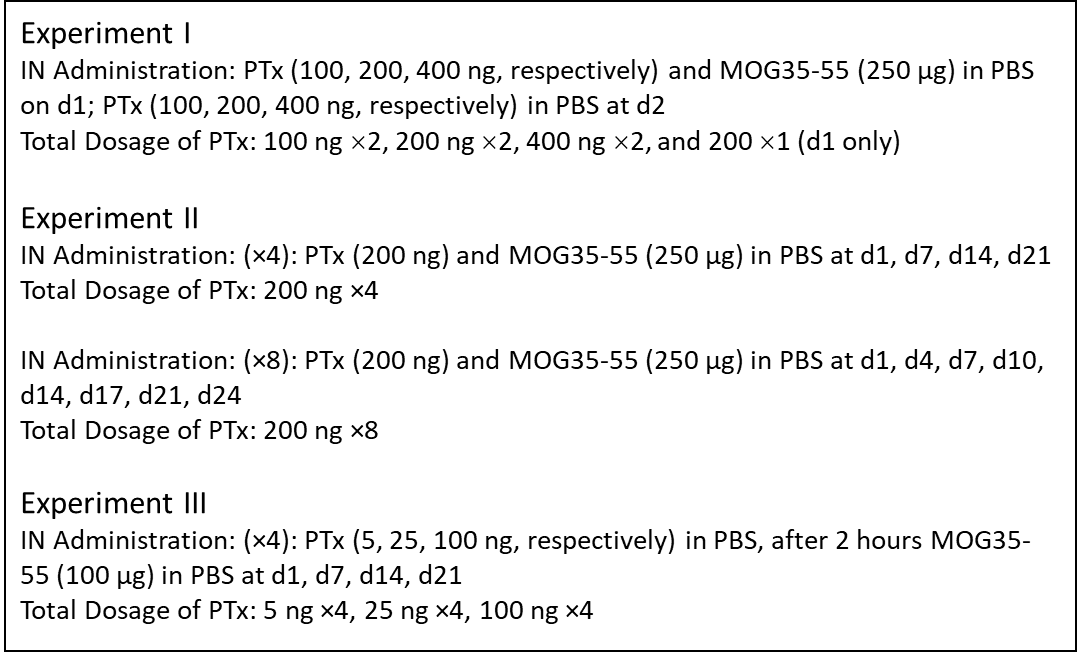


**Supplementary Figure 1.** Experiments performed testing PTx as an adjuvant by intranasal administration. Experiments of I and II were initially performed testing whether to be able to induce EAE with different dosages of PTx and treatment protocols. We observed that neither EAE nor alopecia were induced. Experiment III treatment protocol induced alopecia at 5ng and 25ng of PTx dosage and indicated treatment protocol.

# Supplementary Figure 2


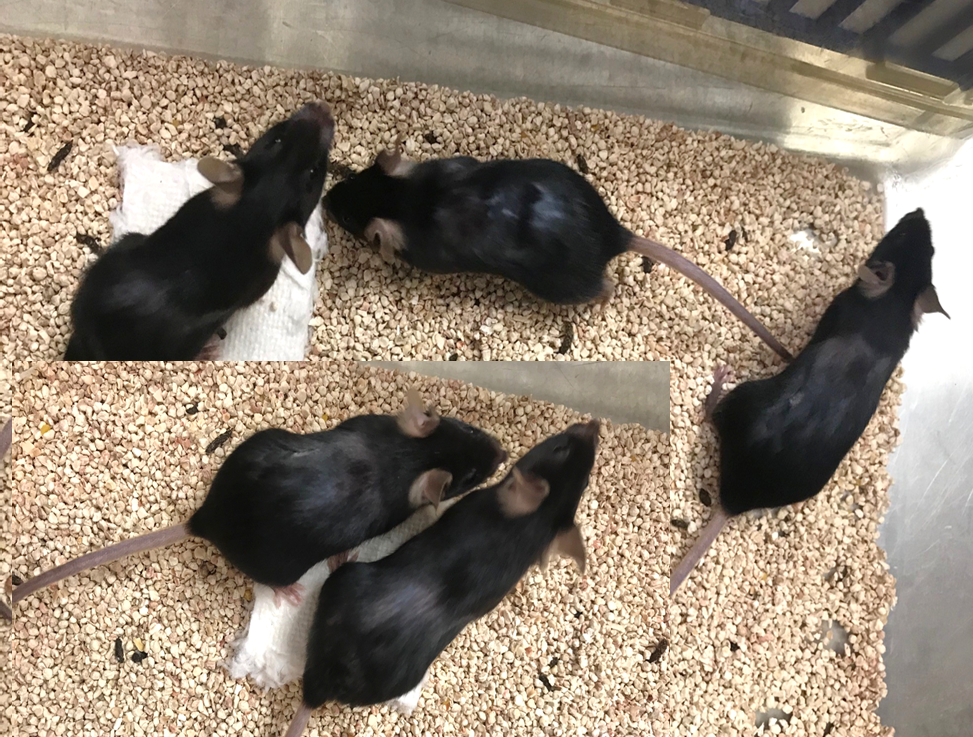


**Supplementary Figure 2.** Mouse images show hair regrowth in mice with alopecia areata. At the end of the observation period, 25% (5/20) of mice with alopecia demonstrated mild-moderate hair regrowth.

# Supplementary Figure 3


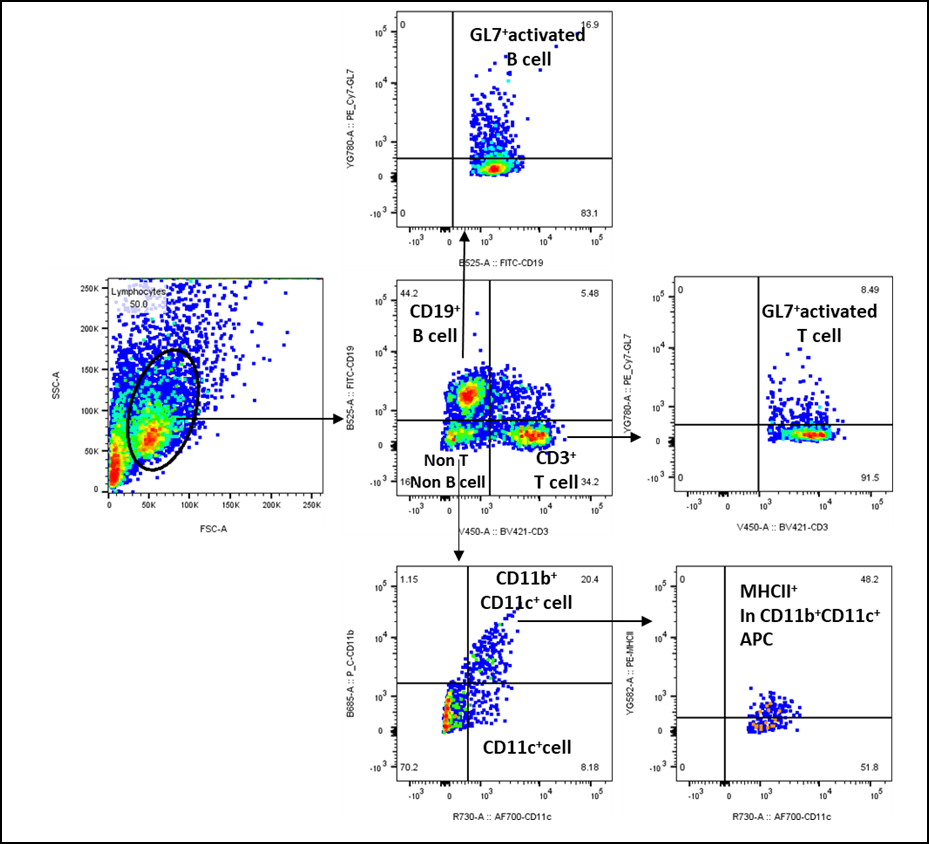


**Supplementary Figure 3.** The gating strategy of immune cells analyzed by flow cytometry. CD3^+^ T and CD19^+^ B cell populations were gated from the defined lymphocyte populations, and T cell and B cell population were further analyzed with antibody to GL7^+^ (for activated T or B cells). Among non-T non-B cell populations, we gated CD11b^+^CD11c^+^ cells and identified MHCII-expressing cells (“APC population”).
